# A Two-Stage Whole-Genome Gene Expression Association Study of Young-Onset Hypertension in Han Chinese Population of Taiwan

**DOI:** 10.1038/s41598-018-19520-w

**Published:** 2018-01-29

**Authors:** Kuang-Mao Chiang, Hsin-Chou Yang, Wen-Harn Pan

**Affiliations:** 1 Institute of Biomedical Sciences, Academia Sinica, Taipei, Taiwan; 2 Institute of Statistical Science, Academia Sinica, Taipei, Taiwan

## Abstract

Hypertension is an important public health problem in the world. Since the intermediate position of the gene expression between genotype and phenotype makes it suitable to link genotype to phenotype, we carried out a two-stage whole-genome gene expression association study to find differentially expressed genes and pathways for hypertension. In the first stage, 126 cases and 149 controls were used to find out the differentially expressed genes. In the second stage, an independent set of samples (127 cases and 150 controls) was used to validate the results. Additionally, we conducted a gene set enrichment analysis (GSEA) to search for differentially affected pathways. A total of nine genes were implicated in the first stage (Bonferroni-corrected p-value < 0.05). Among these genes, *ZRANB1*, *FAM110A*, *PREP*, *ANKRD9* and *LAMB2* were also differentially expressed in an existing database of hypertensive mouse model (GSE19817). A total of 16 pathways were identified by the GSEA. *ZRANB1* and six pathways identified are related to TNF-α. Three pathways are related to interleukin, one to metabolic syndrome, and one to Hedgehog signaling. Identification of these genes and pathways suggest the importance of 1. inflammation, 2. visceral fat metabolism, and 3. adipocytes and osteocytes homeostasis in hypertension mechanisms and complications.

## Introduction

Hypertension is an important public health problem in the world. Essential hypertension is one of the most predictive or associated risk factors of cerebral hemorrhage and infarction, coronary heart disease, heart failure, diabetes mellitus, and kidney disease. The substantial heritability (30–60%)^1–3^ of hypertension has prompted scientists to study its genetic underpinnings through genetic and expression profiling. Findings on hypertension genes can be used not only for screening high-risk individuals, preventing disease development, but also for elucidating disease mechanisms. Most of the previous hypertension genetic study focused on hypertension with relatively older ages. We focused on young-onset hypertension which has a stronger genetic component than hypertension in general^4,5^. In this study, we followed our previous studies which used age 50 as the cut-off to screen young-onset hypertension^4,5^.

Gene expression level may represent the phenotype most immediately connected to DNA sequence variation. The intermediate position of the gene expression makes it suitable to bridge between genotype and phenotype^6^. Recent advances in molecular biology and technology have made it possible to monitor the expression levels of all genes simultaneously. Several gene expression association studies on human hypertension have been conducted^7–10^.

The mechanism of essential hypertension is complex. The target tissues of essential hypertension are likely multiple. Animal studies on hypertension gene expression have used the aorta, heart, liver and kidney tissues^11^, but these tissues are not accessible in human. Human lymphoblastoid cell lines (LCLs) are gaining popularity as a research tool for studying genome-wide individual differences^12,13^. LCL presents some advantages not only in its tractability and availability, but also in its potential of negating environmental influence. Lymphoblast cells are transformed and grown in the same conditions, which presumably minimize the environmental sources of variation^14,15^.

In this study, we conducted a two-stage whole-genome gene expressions association study. Afterwards, a hypertensive mouse data obtained from Gene Expression Omnibus (GEO)^16^ database was used to further support our findings. Moreover, we further conducted a gene set enrichment analysis (GSEA)^17^ to investigate whether the transcripts were enriched for the various known pathways.

## Materials and Methods

### Ethics Statement

Written informed consent was obtained from each participant at his/her initial clinic visit. The study was approved by the Internal Review Board of Academia Sinica (Permit Number: AS-IRB01-08012). All methods were performed in accordance with the relevant ethical guidelines and regulations.

### Study Design

**Figure 1:**
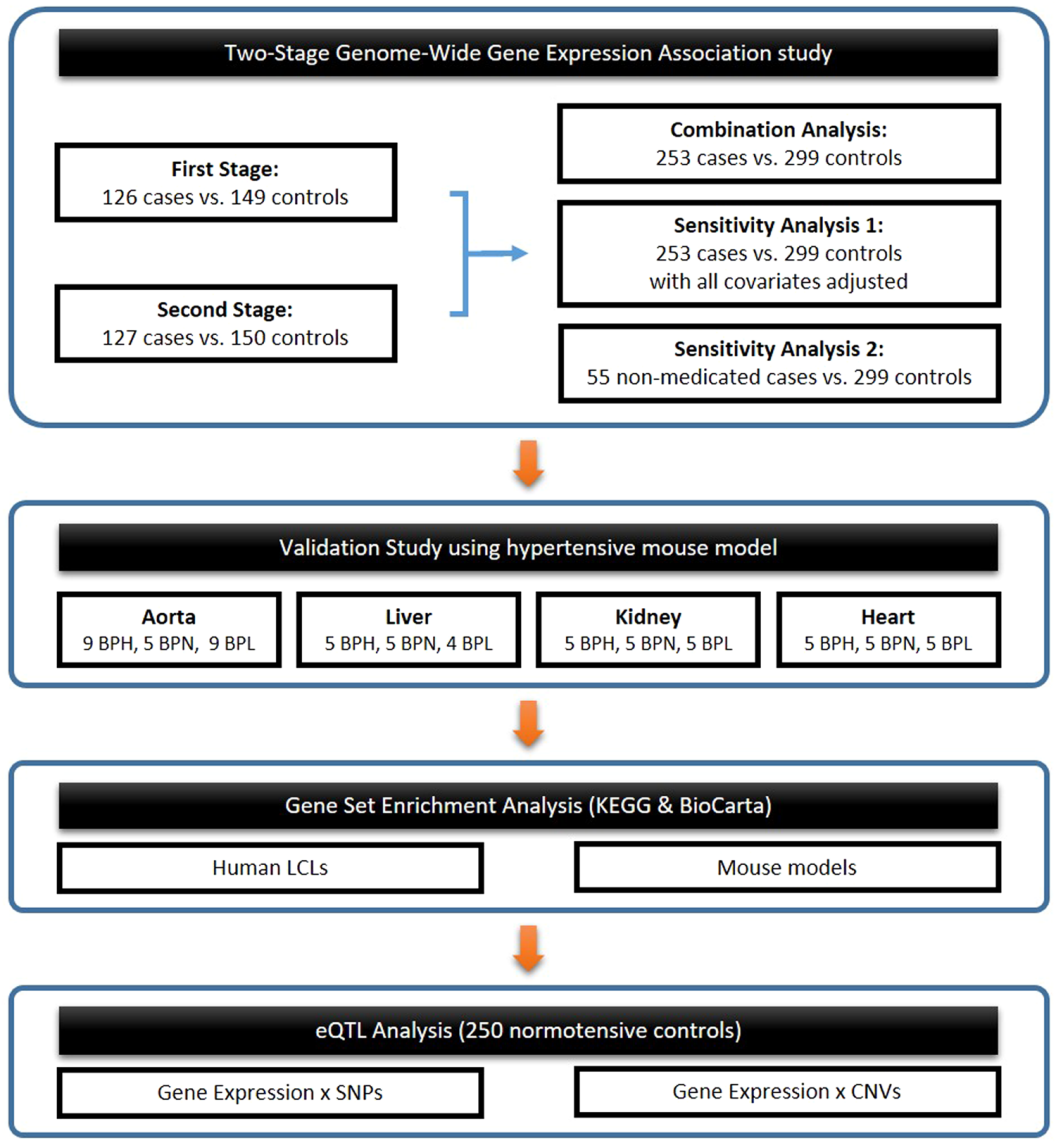
Flow chart of the study design. BPH: high blood pressure, BPL: lowest blood pressure, and BPN: normal blood pressure, LCLs: lymphoblastoid cell lines, eQTL: expression quantitative trait loci, SNP: single nucleotide polymorphism, CNV: copy number variation.

Figure 1 shows the flowchart of the study design. A two-stage whole-genome gene expression association study was carried out in Han Chinese to search for multiple essential hypertension genes with differential gene expression in hypertensive patients and normotensive controls.

In the first stage, a total of 126 young-onset hypertensive patients and 149 normotensive controls were included. In the second stage, another 127 young-onset hypertensive patients and 150 normotensive controls were used to validate the finding in the first stage. Finally, we combined 253 case and 299 control samples together in the combination analysis. The characteristics of these samples are presented in the Tables 1 and 2.

**Table 1:** Characteristics of the participants – continuous traits. BMI: body mass index, TG: triglyceride, HDL-C: high density lipoprotein cholesterol, LDL-C: low density lipoprotein cholesterol, SBP: systolic blood pressure, DBP: diastolic blood pressure. eGFR (mL/min/1.73m^2^) = 175 × (Scr)-1.154 × (Age)-0.203 × (0.742 if female) × (1.212 if African American).

1^st^ Stage	Case (N = 126)		Control (N = 149)		*p-value*
	Mean	(SD)	Mean	(SD)	
Age (year)	38.2	9.1	37.6	9.6	0.57
BMI (Kg/M^2^)	26.5	3.8	23.1	3.4	<0.0001*
Uric acid (mg/dl)	6.9	1.9	6.0	1.8	<0.0001*
Fasting Glucose (mg/dl)	97.7	9.0	—	—	—
HbA1c (%)	—	—	4.7	0.8	—
Total Cholesterol (mg/dl)	199.9	36.5	179.3	40.4	<0.0001*
TG (mg/dl)	155.8	102.2	—	—	—
HDL-C (mg/dl)	47.1	13.9	49.6	12.3	0.11
LDL-C (mg/dl)	130.5	33.9	—	—	—
Creatinine (mg/dl)	0.86	0.18	0.95	0.16	<0.0001*
eGFR (mL/min/1.73 m^2^)	97.1	20.0	85.0	14.7	<0.0001*
s-GOT (U/L)	22.5	8.0	24.9	11.6	0.051
s-GPT (U/L)	27.5	19.5	26.1	26.1	0.60
SBP (mmHg)	125.4	16.4	108.6	9.0	<0.0001*
DBP (mmHg)	84.0	12.6	71.3	7.6	<0.0001*
**2** ^**nd**^ **Stage**	**Case (N = 127)**		**Control (N = 150)**		**p-value**
	**Mean**	**(SD)**	**Mean**	**(SD)**	
Age (year)	37.7	8.7	37.5	10.0	0.88
BMI (Kg/M^2^)	26.2	3.7	23.1	2.9	<0.0001*
Uric acid (mg/dl)	6.6	1.8	6.1	1.7	0.017*
Fasting Glucose (mg/dl)	97.0	9.5	—	—	—
HbA1c (%)	—	—	4.6	0.6	—
Total Cholesterol (mg/dl)	191.5	36.3	176.6	31.1	0.0003*
TG (mg/dl)	152.5	108.5	—	—	—
HDL-C (mg/dl)	46.6	11.4	50.7	12.9	0.0063*
LDL-C (mg/dl)	122.2	31.5	—	—	—
Creatinine (mg/dl)	0.83	0.19	0.92	0.18	<0.0001*
eGFR (mL/min/1.73 m^2^)	99.5	21.8	86.5	15.4	<0.0001*
s-GOT (U/L)	21.3	7.8	25.0	14.6	0.0081*
s-GPT (U/L)	25.7	16.9	24.5	19.5	0.58
SBP (mmHg)	126.8	14.5	109.9	8.3	<0.0001*
DBP (mmHg)	84.9	11.1	71.4	7.2	<0.0001*

**Table 2:** Characteristics of participants – categorical traits.

1^st^ Stage	Case (N = 126)		Control (N = 149)		*p-value*
	N	%	N	%	
**Sex**					0.88
Male	92	73.0	110	73.8	
Female	34	27.0	39	26.2	
**Drinking habit**					<0.0001*
Never	71	56.4	35	23.5	
Seldom	0	0	63	42.3	
Occasionally	40	31.8	11	7.4	
Persistently	15	11.9	40	26.9	
**Smoking habit**					0.028*
Never	94	74.6	87	58.8	
0–5 years	3	2.4	6	4.1	
6–10 years	5	4.0	15	10.1	
11–20 years	5	4.0	16	10.8	
>20 years	19	15.1	24	16.2	
**Education**					0.0043*
Elementary School	7	5.6	10	6.7	
Junior-high/Senior-high	36	28.6	70	47.0	
BS/MS/PhD	83	65.9	69	46.3	
**Diabetes**					—
Yes	0	0	0	0	
No	126	100	145	97.3	
**Antihypertensive medication**					<0.0001*
Yes	101	80.2	0	0	
No	25	19.8	149	100	
**2** ^**nd**^ **Stage**	**Case (N = 127)**		**Control (N = 150)**		***p-value***
	**N**	**%**	**N**	**%**	
**Sex**					0.86
Male	80	63.0	96	64.0	
Female	47	37.0	54	36.0	
**Drinking habit**					<0.0001*
Never	76	59.8	41	27.3	
Seldom	0	0	62	41.3	
Occasionally	38	29.9	19	12.7	
Persistently	13	10.2	28	18.7	
**Smoking habit**					0.064
Never	89	70.1	92	61.3	
0–5 years	3	2.4	13	8.7	
6–10 years	5	3.9	14	9.3	
11–20 years	13	10.2	15	10.0	
>20 years	17	13.4	16	10.7	
**Education**					<0.0001*
Elementary School	3	2.4	21	14	
Junior-high/Senior-high	40	31.5	68	45.3	
BS/MS/PhD	84	66.1	61	40.7	
**Diabetes**					—
Yes	0	0	0	0	
No	127	100	149	100	
**Antihypertensive medication**					<0.0001*
Yes	97	76.4	0	0	
No	30	23.6	150	100	

*p < 0.05.

### Study participants

Young-onset hypertensive patients were selected from “Academia Sinica Multi-Center Young-Onset Hypertension Genetic Study”, which had recruited 1023 non-aboriginal Taiwanese individuals with essential hypertension aged 20 to 50. Details regarding this young-onset hypertension genetic study has been described elsewhere^4,5^. Age and sex matched normotensive controls were selected from “Han-Chinese Cell and Genome Bank in Taiwan”^18^ which has established by Institute of Biomedical Sciences of Academia Sinica.

Inclusion criteria for young-onset hypertensive patients are described as follows: (a) systolic blood pressure (SBP) ≥140 mmHg and/or diastolic blood pressure (DBP) ≥ 90 mmHg at least twice in the previous 2–6 months, or SBP/DBP ≥ 120/80 mmHg at least twice for those who were on anti-hypertensive medication for two months or more; (b) participant initially diagnosed with hypertension between the ages of 20 and 51years; (c) no secondary hypertension such as chronic renal disease, renal arterial stenosis, primary aldosteronism, coarctation of the aorta, thyroid disorders, Cushing’s syndrome and pheochromocytoma (confirmed through extensive clinical investigations including blood chemistry, renal function test, endocrine 6procedures and abdominal sonogram); (d) no medical history regard to severe disease, including liver and renal failure; carcinoma; cardiac or pulmonary failure (e) fasting glucose level < 126 mg/dl and no previous diagnosis of diabetes mellitus; (f) body mass index (BMI) < 35 Kg/m^2^; (g) both parents and all grandparents are Han-Chinese; and (h) have been genotyped by Illumina 550 K beadchip.

Inclusion criteria for normotensive controls are: (a) participant with SBP < 120 mmHg and DBP < 80 mmHg and no anti-hypertensive medication (b) aged between 20 to 51 years; (c) no other disease (d) BMI < 35 Kg/m^2^; (e) both parents and all grandparents are Han-Chinese; and (f) have been genotyped by Illumina 550 K beadchip. Finally, age and sex matched controls were selected to carry out the following experiments.

### Establishing Lymphoblastoid Cell Lines

Lymphoblastoid Cell Lines (LCLs) were used to profile the gene expression levels. A total of 33 ml of blood had been drawn from each eligible subject: 17.5 ml for basic clinical chemistry and plasma storage, 5 ml for DNA extraction, 10 ml for LCLs establishment, and 0.5 ml for Guthrie cards.

Peripheral blood samples were collected and LCLs were generated by Epstein Barr Virus transformation of the B-lymphocyte component by the Bioresource Collection and Research Center (BCRC, Hsinchu, Taiwan) and transformed cells were grown in RPMI 1640 (Invitrogen, Carlsbad, CA) with 15% fetal bovine serum (FBS, Hyclone, Logan, UT), 100 U/ml penicillin and 100 g/ml streptomycin (Sigma, St. Louis, MO).

The LCLs of all cases and controls used in 1^st^ stage (126 cases and 149 controls) and 2^nd^ stage (127 cases and 150 controls) were generated in the same time period in the same laboratory.

### RNA Extraction and Gene Expression Profiling

For the gene expression profiling, the case-control pair was assayed back to back to avoid differential expression level caused by batch effect.

Total RNA was extracted with TRIzol (Invitrogen) and hybridized onto Human OneArray v5.1 (HOA v5.1, Phalanx Biotech. Group, Hsinchu, Taiwan). For the whole genome gene expression profile measurement, only the RNA which RNA Integrity Number (RIN) > 7 were used.

Phalanx Human OneArray v5.1 contains 30,968 human genome probes. Each sample was measured with 3 technical repeats (1,656 arrays, 552 samples x 3 repeats). A microarray data management and analysis software, BRB Array tools^19^ and R statistical software were employed for quality control of the data. The data quality control procedures including: (1) background correction (subtracting the average value of the negative control probes), (2) removal of 5,752 probes with detection p-value > 0.05, (3) quantile normalization for all 1,656 arrays, (4) removal of samples for which 3 replicated samples were not clustered for the remaining 25,216 probes, (5) median value of 3 replicates selected for further analysis, and (6) use of ComBat^20^, an empirical Bayes method (R package: SVA), to remove remaining batch effects.

### Two-Stage Whole-Genome Gene Expression Association Study

In the first stage (126 case and 149 controls), logistic regression with age, sex and BMI adjustment was used to identify the significantly differentially expressed genes with hypertension status (0, 1) as the independent variable. In the second stage, another independent sample set (127 cases and 150 controls) was used to validate the transcripts found in the first stage. The same statistical methods were used with age, sex, and BMI adjustment. Finally, in the combination analysis, we combined samples of the two stages together (253 cases and 299 controls) for the association test and calculated the fold change (average intensities in cases/average intensities in controls). The same analysis methods employed in the first and stages were used in the combination analysis. Bonferroni correction was employed in the first stage, second stage and combination analysis for handling multiple comparison issue. The commercial software SAS 9.4 was used to do the statistical analyses.

### Sensitivity Analysis

We used the combined samples to conduct a sensitivity analysis which included the age, sex, BMI, liver function (s-GOT & s-GPT), total-cholesterol (TC), HDL-C, kidney function (eGFR), serum uric acid, education, smoking and alcohol use in the model to check whether the results are robust to these factors.

Besides, we also performed a sensitivity analysis among the non-medicated patients to confirm that these transcripts were not due to effect of antihypertensive medications. Among the 253 young-onset hypertensive patients, 55 patients were non-medicated. We used these 55 non-medicated patients and 299 healthy controls to perform the sensitivity analysis.

### Validation study using hypertensive mouse data

Furthermore, we used gene expression profile data of hypertensive mouse model downloaded from the Gene Expression Omnibus (GEO, GSE19817, http://www.ncbi.nlm.nih.gov/geo/GPL9734)^11^ to validate these results.

Male BPH/2J (high blood pressure), BPL/1J (lowest blood pressure), and BPN/3J (normal blood pressure) mice were used to generate expression data. Gene expression profiles of tissues including aorta (9 BPH, 5 BPN and 9 BPL), liver (5 BPH, 5 BPN and 4 BPL), heart (5 BPH, 5 BPN and 5 BPL), and kidney (5 BPH, 5 BPN and 5 BPL) were measured. Merck/Affymetrix mouse 1.0 custom arrays monitoring 38,384 individual transcripts (25846 Entrez genes) were used. Raw intensity was normalized using the RMA algorithm^21^. BRB-Arraytools was used to analyze the normalized data with 10,000 permutations to identify the differential expressed ones among the identified genes. FDR was employed to control the multiple comparisons.

### Gene Set Enrichment Analysis (GSEA)

On top of the two-stage whole-genome gene expression association study, we further conducted a gene set enrichment analysis (GSEA) to investigate the differentially expressed pathways. BRB-array tools^19^ was employed to perform the analysis. A total of 299 pathways from BioCarta database and 128 pathways from KEGG database were tested. For the GSEA, all of the 25,216 transcripts were included. Two-sample *t*-test was used to get the p-values from each single transcript. Least square (LS) permutation test and Kolmogorov–Smirnov (KS) permutation test were used to find significant gene sets. LS/KS permutation test finds gene sets which have more genes differentially expressed among the phenotype classes than expected by chance^22^. These results were further validated using hypertensive mouse data.

### Expression quantitative trait loci (eQTL) Analysis

We integrated the gene expression profiles with the SNP and copy number variation (CNV) data to find influential genetic polymorphisms for the differentially expressed genes. Use was made of data from 250 (out of 299) normotensive controls, who had both genotype and gene expression data.

These samples were genotyped with the Illumina’s Sentrix^®^ HumanHap550 Genotyping BeadChip which contains 560,184 tag-SNPs. Genomic DNA was isolated from leukocytes using a PUREGENE® DNA Purification Kit (QIAGEN®, Gentra Systems, Minneapolis, MN, USA) for genomic DNA isolation. The DNA concentration was quantified and adjusted to 60 ng/μl using a NanoDrop™ ND-1000 Spectrophotometer (NanoDrop™ Technologies, DE, USA). All sample were genotyped by deCODE Genetics (Reykjavík, Iceland).

Genotype calling was performed using the standard procedure implemented in BeadStudio (Illumina®, Inc., San Diego, CA, USA) with the default parameters.

Information on genotyping call rate (GCR), Hardy-Weinberg Equilibrium (HWE) and minor allele frequency (MAF) was used to evaluate Genotyping quality. SNPs were excluded, if: (1) they were nonpolymorphic in both cases and controls, (2) GCR < 0.95, (3) MAF < 0.01, or (4) SNP deviated from HWE with a -log10(pFDR) > 3 (where FDR is false discovery rate). Finally, 497,849 SNPs (~88.9% of SNPs on the HumanHap550 BeadChip) were analyzed in this study.

Genotype based (AA, AB, BB) regression was employed to test the association between genotype and gene expression. Only cis-SNPs (SNPs located in the gene region ± 50 Kbps) were used to test the association. We then tested the association between the discovered expression regulatory SNPs (eSNPs) and young-onset hypertension. Statistical significance was claimed under a significance threshold of p-value < 0.05.

Furthermore, the intensity data of 497,849 SNPs were used to call the CNV regions. CNVs were identified using PennCNV^23^ and QuantiSNP^24^, respectively, which identifies CNVs by integrating intensity data from neighboring probes using a hidden Markov model (HMM). Gene-based CNVs association analysis was used to test whether there was a significantly differed probability of a CNV intersecting a given gene between hypertension patients and normotensive controls. CNV analysis were performed in PLINK^25^.

## Results

Tables 1 and 2 lists the continuous and categorical characteristics traits, respectively, of the young-onset hypertension cases and normotensive controls. Overall, patients had higher BMI, uric acid (UA), total cholesterol (TC), eGFR, alcohol and cigarette use, education level and lower HDL-C level.

### Two-stage Whole-genome Gene Expression Association Study

In the first stage, 126 young-onset hypertension cases and 149 normotensive controls were included. Logistic regression model was employed with age, sex, and BMI adjustment. After Bonferroni correction (p-value < 1.98 × 10^−6^, 0.05/25215), 9 transcripts (*NRG2*, *DCPS*, *ZRANB1*, *PREP*, *ANKRD9*, *WFDC12*, *TPTE*, *FAM110A*, and *LAMB2*) were considered significantly differentially expressed (Table 3 and Figure S1).

**Table 3:** Results of the 1st stage and 2nd stage gene expression association studies. *Bonferroni = (p-value x 25215); Chr: Chromosome; SD: Standard deviation; Fold Change = (average intensities in cases)/(average intensities in controls); OR: Odds ratio; CI: Confidence interval.

Chr	Gene	Probe ID	1^st^ stage (126 cases: 149 controls)		2^nd^ stage (127 cases: 150 controls)		Combination Analysis (253 cases: 299 controls)							
			p-value	Bonferroni	p-value	Bonferroni	Intensities in Cases		Intensities in Controls		Fold	p-value	Bonferroni	OR
							Mean	(SD)	Mean	(SD)	Change			(95% CI)
10	*ZRANB1*	PH_hs_0002321	6.83 × 10^−10^	1.72 × 10^−5^	4.91 × 10^−7^	1.24 × 10^−2^	549.85	−191.99	724.7	−194.32	0.76	1.85 × 10^−15^	4.66 × 10^−11^	0.12 (0.07, 0.20)
11	*DCPS*	PH_hs_0029599	3.99 × 10^−8^	1.01 × 10^−3^	2.21 × 10^−8^	5.57 × 10^−4^	877.73	−194.19	641.45	−257.65	1.37	3.95 × 10^−15^	9.96 × 10^−11^	9.98 (5.62, 17.71)
5	*NRG2*	PH_hs_0028351	1.70 × 10^−7^	4.29 × 10^−3^	1.05 × 10^−7^	2.65 × 10^−3^	139.77	−145.56	92.27	−26.69	1.51	9.05 × 10^−14^	2.28 × 10^−9^	4.47 (3.02, 6.61)
2	*PREP*	PH_hs_0025301	4.57 × 10^−7^	1.15 × 10^−2^	3.14 × 10^−8^	7.92 × 10^−4^	904.81	−171.69	671.62	−276.36	1.35	5.00 × 10^−14^	1.26 × 10^−9^	8.81 (5.00, 15.52)
20	*WFDC12*	PH_hs_0032804	5.60 × 10^−7^	1.41 × 10^−2^	6.90 × 10^−7^	1.74 × 10^−2^	114.19	−82.83	87.91	−21.05	1.3	1.59 × 10^−12^	4.01 × 10^−8^	5.20 (3.29, 8.21)
20	*FAM110A*	PH_hs_0035375	7.20 × 10^−7^	1.82 × 10^−2^	7.20 × 10^−7^	1.82 × 10^−2^	353.95	−516.43	261.3	−70.34	1.35	8.64 × 10^−10^	2.18 × 10^−5^	3.83 (2.50, 5.87)
3	*LAMB2*	PH_hs_0025765	1.24 × 10^−6^	3.13 × 10^−2^	1.24 × 10^−6^	3.13 × 10^−2^	29.57	−15.25	24.6	−4.31	1.2	4.77 × 10^−10^	1.20 × 10^−5^	7.38 (3.94, 13.85)
21	*TPTE*	PH_hs_0027797	1.60 × 10^−6^	4.0.3 × 10^−2^	7.84 × 10^−7^	1.98 × 10^−2^	17.77	−14.43	14.21	−2.72	1.25	6.04 × 10^−12^	1.52 × 10^−7^	10.12 (5.23, 19.58)
14	*ANKRD9*	PH_hs_0042480	1.79 × 10^−6^	4.51 × 10^−2^	3.65 × 10^−8^	9.20 × 10^−4^	167.1	−75.37	112.77	−74.28	1.48	3.26 × 10^−13^	8.22 × 10^−9^	3.29 (2.39, 4.53)

In the second stage, another independent sample consisting set of 127 cases and 150 normotensive controls were used to confirm the nine genes that discovered by first stage. All of these nine genes were significantly associated with hypertension in the second stage. According to the Table 3 and the heatmap (Figure S2), except for *ZRANB1* gene (fold change = 0.76; OR = 0.12), most genes were highly expressed in case group compared to the healthy control group. Although the fold change of these up-regulated genes are not very big (ranged from 1.20 to 1.51), the p-value and OR of these genes are strong (OR = 3.29 to 10.12), which means the variance of these genes’ expression are small and the relative risk estimates are rather precise.

Moreover, we have conducted a sensitivity analysis using all 253 case and 299 controls, which included the liver function (GOT & GPT), total-cholesterol, HDL-C, eGFR, SUA, smoking, alcohol consumption, education as well as age, sex and BMI in the model. After adjustment of these covariates, these nine genes still significantly associated with young-onset hypertension (Table S1 in the Supplemental Materials).

We have also performed a sensitivity analysis among the non-medicated individuals to confirm that these transcripts were not due to effect of antihypertensive medications. The association between the genes and diseases status are still very significant, which demonstrate that our results were not affected by the antihypertensive medication (Table S2 in the supplemental materials).

### Validation Study in a Hypertensive Mouse Model

Furthermore, we used the mouse gene expression profile data downloaded from GEO (GSE19817)^11^ to validate these results. This data profiled gene expression in liver, heart, kidney, and aorta from the genetically hypertensive “blood pressure high” (BPH), normotensive “blood pressure normal” (BPN), and hypotensive “blood pressure low” (BPL) inbred mouse strains. Among these nine genes, *Nrg2* cannot be found on this custom array and was not included in the following analyses.

After 10,000 times permutation along with the FDR correction (pFDR < 0.05), *Zranb1* can be validated in the aorta, liver and kidney (pFDR = 0.0157, 0.048 and 0.0341, respectively). *Fam110a* is significantly differentially expressed in aorta and kidney (pFDR = 8.8 × 10^−6^ and 0.034, respectively). *Prep*, *Ankrd9* and *Lamb2* can be only validated in aorta (pFDR of *Prep* = 0.016), liver (pFDR of *Ankrd9* = 0.048) and kidney (pFDR of *Lamb2* = 0.034), respectively. Although the raw p-value of *Dcps* in Heart (p = 0.0084)*, Wfdc12* in aorta (p = 0.037) and liver (p = 0.04) and *Tpte* in liver (p = 0.045) were smaller than 0.05, none of these genes were significant after FDR correction. The results have been shown in Table 4.

**Table 4:** Results of validation study in the hypertensive mouse model.

Chr	Gene	Tissue	p-value	FDR	Mean of intensities in class 1	Mean of intensities in class 2	Mean of intensities in class 3	Pairwise significant
2	Prep*	Aorta	0.0043	0.016*	334.71	393.88	384.11	(1, 3), (1, 2)
		Heart	0.0192	0.071	342.5	402.2	375.42	(1, 2)
3	Lamb2*	Kidney	0.0034	0.0341*	130.68	121.49	143.28	(2, 3)
10	Zranb1*	Aorta	0.0039	0.016*	381.84	568.03	474.53	(1, 2)
		Liver	0.012	0.048*	88.44	66.53	81.23	(2, 1)
		Liver	0.013	0.048*	270.59	238.56	287	(2, 3)
		Kidney	0.0062	0.034*	239.29	210.93	248.18	(2, 3)
11	*Dcps*	Heart	0.0084	0.067	213.73	192.58	228.67	(2, 3)
14	Ankrd9*	Liver	0.012	0.048*	25.76	30.01	23.61	(3, 2)
20	Fam110a*	Aorta	8.00E-07	0.000009*	207.65	128.85	122.62	(3, 1), (2, 1)
		Heart	0.012	0.067	102.09	103.47	92.19	(3, 2)
		Kidney	0.035	0.13	179.28	153.71	152.83	—
20	*Wfdc12*	Aorta	0.037	0.10	21.95	18.43	18.01	—
		Liver	0.040	0.097	10.41	11.33	10.12	—
21	*Tpte*	Liver	0.045	0.097	10.29	11.55	10.74	—

FDR: False discovery rate; *FDR < 0.05. Only the genes which p-value < 0.05 were shown in this table.

Class 1: Hypotensive mouse model, class 2: Normotensive mouse, class 3: Hypertensive mouse.

### Gene Set Enrichment Analysis

In addition to the whole-genome gene expression association study, we also conducted a gene set enrichment analysis (GSEA) to investigate whether any known pathway maybe implicated. Table 5 showed the GSEA results of BioCarta database and KEGG database. Only the pathways that reached the significant threshold (p-value < 0.05) in the both LS permutation and KS permutation simultaneously were shown. There were 14 pathways identified from BioCarta, and 2 pathways identified from KEGG, respectively.

**Table 5:** Results of pathway analysis.

No.	Pathway	Pathway description	Gene No.	LS permutation p-value	KS permutation p-value
1	h_tnfr1Pathway	*TNFR1 Signaling Pathway*	40	***0.0058***	***0.040***
2	h_pmlPathway	*Regulation of transcriptional activity by PML*	27	***0.0069***	***0.0024***
3	h_HivnefPathway	*HIV-I Nef: negative effector of Fas and TNF*	73	***0.0082***	***0.023***
4	h_stressPathway	*TNF/Stress Related Signaling*	33	***0.011***	***0.0046***
5	h_tidPathway	*Chaperones modulate interferon Signaling Pathway*	22	***0.012***	***0.018***
6	h_malPathway	*Role of MAL in Rho-Mediated Activation of SRF*	25	***0.020***	***0.021***
7	h_arenrf2Pathway	*Oxidative Stress Induced Gene Expression Via Nrf2*	29	***0.020***	***0.015***
8	h_biopeptidesPathway	*Bioactive Peptide Induced Signaling Pathway*	40	***0.021***	***0.0070***
9	h_il2Pathway	*IL 2 signaling pathway*	30	***0.028***	***0.026***
10	h_no2il12Pathway	*NO2-dependent IL 12 Pathway in NK cells*	20	***0.029***	***0.0030***
11	h_il10Pathway	*IL-10 Anti-inflammatory Signaling Pathway*	19	***0.044***	***0.011***
12	h_vobesityPathway	*Visceral Fat Deposits and the Metabolic Syndrome*	10	***0.045***	***0.016***
13	h_hsp27Pathway	*Stress Induction of HSP Regulation*	16	***0.046***	***0.0084***
14	h_atmPathway	*ATM Signaling Pathway*	25	***0.049***	***0.036***
15	hsa00533	*Keratan sulfate biosynthesis*	17	***0.0085***	***0.016***
16	hsa04340	*Hedgehog signaling pathway*	79	***0.015***	***0.0029***

LS permutation: Least square permutation; KS permutation: Kolmogorov–Smirnov permutation. The LS/KS permutation tests, which find gene sets that have more differentially expressed genes among the classes than expected by chance.

Furthermore, we also used the mouse gene expression profile data to validate these results. Five out of 16 pathways can be validated in the mouse model (Table 6).

**Table 6:** Validation results of pathway analysis based on mouse data.

Tissues/No.	Pathway	Pathway description	Gene No.	LS permutation p-value	KS permutation p-value
**Aorta**					
1	m_arenrf2Pathway	*Oxidative Stress Induced Gene Expression Via Nrf2*	24	***0.045***	***0.016***
**Heart**					
1	m_il10Pathway	*IL-10 Anti-inflammatory Signaling Pathway*	13	***0.004***	***0.016***
2	m_arenrf2Pathway	*Oxidative Stress Induced Gene Expression Via Nrf2*	24	***0.009***	***0.002***
3	m_biopeptidesPathway	*Bioactive Peptide Induced Signaling Pathway*	38	***0.021***	***0.001***
4	m_malPathway	*Role of MAL in Rho-Mediated Activation of SRF*	28	***0.027***	***0.005***
**Liver**					
1	m_biopeptidesPathway	*Bioactive Peptide Induced Signaling Pathway*	38	***0.015***	***0.002***
2	m_HivnefPathway	*HIV-I Nef: negative effector of Fas and TNF*	77	***0.023***	***0.002***
3	m_arenrf2Pathway	*Oxidative Stress Induced Gene Expression Via Nrf2*	24	***0.035***	***0.003***
**Kidney**					
1	m_biopeptidesPathway	*Bioactive Peptide Induced Signaling Pathway*	38	***0.003***	***0.008***

LS permutation: Least square permutation; KS permutation: Kolmogorov–Smirnov permutation. The LS/KS permutation tests, which find gene sets that have more differentially expressed genes among the classes than expected by chance.

### Data availability

For future meta-analysis, our information on all p-values data are available for download from the website: http://pan.ibms.sinica.edu.tw/hypertension/data3.

## Discussion

In this study, a total of nine genes were found differentially expressed in two independent sample sets. Among these genes, five genes (*ZRANB1*, *FAM110A*, *PREP*, *ANKRD9* and *LAMB2*) were identified and replicated by our study, and also were validated in the hypertensive mouse model. *NRG2*, *DCPS*, *WFDC12*, and *TPTE* were identified and replicated by our study, but not replicated in the hypertensive mouse model. The discrepancy may be due to the different tissues used in our study (LCLs) and in the mouse model (aorta, heart, liver, and kidney) or due to the species specificity (human and mouse).

The down-regulated gene, *ZRANB1*, is playing a role in the regulation of cell morphology and cytoskeletal organization and being required in the stress fiber dynamics and cell migration. This gene may also modulate TNF-alpha signaling which has been associated with hypertension^26^. *NRG2* encodes a novel member of the neuregulin family of growth and differentiation factors^27^. Through interaction with the ErbB family of receptors, this protein induces the growth and differentiation of epithelial, neuronal, glial, and other types of cells. ErbB family members are implicated in the development of end organ damage, as occurs in hypertension^28^. The mechanism of *FAM110A*, *PREP, ANKRD9, DCPS*, *WFDC12*, *TPTE*, and *LAMB2* genes on the occurrence and development of hypertension is not yet clear. Among these nine genes, except for *NRG2*, the other genes are not included in any known pathway.

We have used the combined samples to conduct a sensitivity analysis, which adjusted for the all covariates, to check whether the results are robust to these factors. After adjustment of these covariates, these nine genes still significantly associated with hypertension (Table S1 in the supplemental materials). We noted that there are differences in ORs from the main findings for some genes. According to Tables 1 and 2, besides the metabolic related traits, the distributions of alcohol consumption, smoking and education are also strikingly different between cases and controls. Since these three covariates all have genetic determinants, some of the genes we found might be related to alcohol consumption, smoking, or education. Therefore, we used the combined sample to check the associations between these three covariates and nine genes and found that *ANKRD9* and *NRG2* is slightly associated with education and smoking (Table S6 in the supplemental materials). The differences in the distributions of alcohol consumption, smoking, and education between cases and controls may be partly due to the effects of these genes. More study is needed to examine the relationships among these genes, covariates and hypertension.

Moreover, we have also performed a sensitivity analysis among the non-medicated individuals to confirm that these transcripts were not due to effect of antihypertensive medications. The association between the genes and diseases status are still very significant, which demonstrate that our results were not affected by the antihypertensive medication (Table S2 in the Supplemental Materials). However, we observed that there are also bigger differences in some ORs of genes (eg. *NRG2, FAM110A, LAMB2* and *TPTE*) when the cases are restricted to individuals not on antihypertensive therapy. It may be due to the different sample size or the different disease stage. These non-medicated patients may have relatively less severe hypertension. These genes might have the different effects contribute to the different hypertension stages. Further study is needed to understand the pathogenesis of hypertension.

In addition to the whole-genome gene expression association study that tested these transcripts one at a time, we performed a gene set enrichment analysis (GSEA) to identify pathways with significantly greater number of genes been down- or up-regulated, compared to the controls. In the GSEA, 16 pathways from BioCarta or KEGG were identified.

Among these pathways, six pathways (pathway No. 1 to 5 and 13 in the Table 5), are all related to the TNF (Tumor necrosis factors) which has been implicated in the development of salt-sensitive hypertension induced by angiotensin II^29^. *ZRANB1*, which identified in the two-stage association study, is also related to the TNF-alpha, but this gene is not involved in any known pathway as yet. Pathway *Bioactive peptide induced signaling pathway* (No. 8 in the Table 5) has already known to relate to the blood pressure regulating peptide angiotensin^30^. Three pathways (No. 9 to 11 in the Table 5) are implicated with the interleukin. Hypertension patients have been shown to have an altered profile of these pro- and anti- inflammatory cytokines^31^. Pathway No. 12 (*Visceral fat deposits and the metabolic syndrome*) is a pathway related to the glucocorticoid receptor, which will activate/inactivate the lipoprotein lipase, TNF-alpha, and insulin resistance^32^. The association between Insulin resistance and hypertension has been well recognized. Up to 80% patients with type 2 diabetes have hypertension^33^. Pathway *hedgehog signaling pathway* (No.16 in the Table 5) is an active pathway during embryogenesis. three hedgehog (HH) gene homologs were discovered in vertebrates; Desert (DHH), Indian (IHH), and Sonic (SHH). SHH is the most widely expressed in adult tissues. The *hedgehog signaling pathway* plays a crucial role in the angiogenesis and vascular remodeling. Hypoxia has been demonstrated to activate the SHH pathway to enhances the progress of vascular remodeling in a number of human diseases, including atherosclerosis and pulmonary artery hypertension^34^. Besides, recent studies have shown that this pathway is linked with some age-related diseases such as metabolic syndrome^35^ which may also relate to the blood pressure.

Pathway data in the KEGG and in the BioCarta are heterogeneous due to the differences in pathway construction purpose and methods^36^. For the same pathways in the KEGG and the BioCarta, not only numbers of genes involved are different, but sometimes with very little overlap. That is why the results of gene set analysis are so different, employing the information provided by the two pathway datasets.

In addition, we have also tried to integrate the single nucleotide polymorphisms (SNPs) and copy number variations (CNVs) data and gene expression profile data to investigate whether these hypertension related genes’ expression levels were regulated by SNPs or CNVs.

We found some expression regulatory SNPs (eSNPs) that are associated with the gene expression level of the *NRG2* (rs2916092) and *PREP* (rs1051484, rs1078726, rs10871983, rs1149305, rs1149309, rs1149313, rs11758609, rs1190050, and rs1190053) (Table S3 in the supplemental materials), but these eSNPs were not associated with the young-onset hypertension in our previous two-stage GWAS^5^ (Table S4 in the supplemental materials). The lack of association between these eSNPs and hypertension may be due to the complicated mechanisms of hypertension and small effects of individual genes. A larger sample size or alternative approaches are needed to show the connection between eSNPs and hypertension. Beside the eSNPs, there are no CNVs associated with these genes’ expression levels (Table S5 in the supplemental material).

The mechanisms contributing to essential hypertension are complex. The target tissues of essential hypertension are likely multiple^11^, but these tissues are not accessible in human. In this study, LCLs were used in all gene expression association tests. The magnitude of expression profiles shared among the different tissues is still under debate. Current estimates are range from very small^37^ to 70–80%^38^. The MuTHER study which has the relative large sample size has demonstrated that around 30% of gene expression profiles are shared among tissues, while 29% are exclusively tissue-specific^12,39^. Therefore, some tissue-specific signals may have been missed, but the findings should be valid.

Several gene expression association studies on human hypertension have been conducted^7–10^. There are no overlapping genes were founded among our single gene analysis and previous studies. It may be due to the different sample size and the different surrogate tissues used. The sample size of the most gene expression studies on hypertension is small (n = 18~20), except a large scale gene expression integrative network analysis which used 3,679 non-medicated individuals in the Framingham Heart Study. Although the identified genes are not overlapping among these studies, all of these studies have identified the genes that related to immune or inflammation. Moreover, in our pathway analysis, we have identified the *IL-10 Anti-inflammatory Signaling Pathway* which is in line with the findings on IL-10R gene by Chon’s hypertension gene expression study^8^.

Our study identified several previously unknown young-onset hypertension genes and pathways in Han Chinese. Identification of these genes and pathways suggest the importance of 1. inflammation, 2. visceral fat metabolism and 3. adipocytes and osteocytes homeostasis in either hypertension etiology or complications. These finding may broaden our understanding of hypertension etiology and major outcome development.

## Electronic supplementary material

Supplementary information
