# Supplementary material for: A Two-Stage Whole-Genome Gene Expression Association Study of Young-Onset Hypertension in Han Chinese Population of Taiwan
Source: Sci Rep. 2018 Jan 29;8:1800. doi: 10.1038/s41598-018-19520-w (PMC5789005; doi:10.1038/s41598-018-19520-w)
Supplement: Supplementary file 1 — Supplementary information [file 41598_2018_19520_MOESM1_ESM.pdf]

A Two-Stage Whole-Genome Gene Expression Association Study of Young-Onset Hypertension in Han Chinese Population of Taiwan

Kuang-Mao Chiang, PhD[a]; Hsin-Chou Yang, PhD[b]; Wen-Harn Pan, PhD[a]*

a. Institute of Biomedical Sciences, Academia Sinica, Taipei, Taiwan

b. Institute of Statistical Science, Academia Sinica, Taipei, Taiwan

SUPPLEMENTARY MATERIALS

Table S1. Results of sensitivity analysis that association test with all covariates (253 cases and 299 controls).

Gene	Probe ID	Chr	Start (bp)	End (bp)	p-value	OR	95% CI
ZRANB1	PH_hs_0002321	10	124,916,899	124,988,189	$4.00x10^{-8}$	0.09	(0.04, 0.21)
DCPS	PH_hs_0029599	11	126,303,752	126,345,754	$2.50x10^{-7}$	10.79	(4.37, 26.64)
NRG2	PH_hs_0028351	5	139,846,779	140,043,437	$2.40x10^{-7}$	4.73	(2.62, 8.53)
PREP	PH_hs_0025301	6	105,277,565	105,403,124	$1.79x10^{-6}$	8.85	(3.61, 21.67)
WFDC12	PH_hs_0032804	20	45,123,425	45,124,465	$1.00x10^{-7}$	6.97	(3.41, 14.25)
FAM110A	PH_hs_0035375	20	833,697	857,463	$1.86x10^{-6}$	5.63	(2.77, 11.46)
LAMB2	PH_hs_0025765	3	49,121,114	49,133,166	$2.91x10^{-5}$	7.92	(3.00, 20.91)
TPTE	PH_hs_0027797	21	10,521,514	10,606,270	$2.20x10^{-7}$	15.39	(5.47, 43.25)
ANKRD9	PH_hs_0042480	14	102,501,760	102,509,799	$5.05.x10^{-5}$	2.65	(1.65, 4.24)

Covariates, including age, sex, BMI, education, smoking, alcohol consumption, total cholesterol, HDL-C, s-GOT, s-GPT, eGFR, and serum uric acid, were adjusted in the logistic regression model; Chr: chromosome; OR: odds ratio; CI: confidence interval.

Table S2. Results of sensitivity analysis which using 55 non-medicated patients and 299 normotensive controls.

Gene	Probe ID	Chr	p-value	OR	95% CI
ZRANB1	PH_hs_0002321	10	$1.80x10^{-7}$	0.14	(0.07, 0.30)
DCPS	PH_hs_0029599	11	$4.88x10^{-6}$	9.21	(3.56, 23.87)
NRG2	PH_hs_0028351	5	$7.30x10^{-10}$	8.93	(4.45, 17.91)
PREP	PH_hs_0025301	6	$1.12x10^{-5}$	7.72	(3.10, 19.21)
WFDC12	PH_hs_0032804	20	$2.50x10^{-6}$	6.48	(2.98, 14.10)
FAM110A	PH_hs_0035375	20	$1.25x10^{-6}$	5.97	(2.90, 12.29)
LAMB2	PH_hs_0025765	3	$6.34x10^{-6}$	14.12	(4.47, 44.58)
TPTE	PH_hs_0027797	21	$2.00x10^{-8}$	29.10	(8.96, 94.56)
ANKRD9	PH_hs_0042480	14	$2.68x10^{-5}$	2.98	(1.79, 4.96)

Patients with antihypertensive medication were removed from the sensitivity analysis; Age, sex and BMI were adjusted in the logistic regression model; Chr: chromosome; OR: odds ratio; CI: confidence interval.

Table S3. eSNPs of the Genes identified in the 1st and 2nd stages.

Gene	SNP	Chr	Position	Genotype Based P-Value	Genevar
NRG2	rs2916092	5	139394936	**0.0372***	NS
	rs1051484	6	105832729	**0.0007***	NS
	rs1078726	6	105919333	**0.0018***	**0.027***
	rs10871983	6	105942355	**0.0020***	**0.014***
	rs1149305	6	105852507	**0.0029***	**0.026***
PREP	rs1149309	6	105856616	**0.0032***	**0.019***
	rs1149313	6	105860999	**0.0157***	NS
	rs11758609	6	105948645	**0.0160***	**0.046***
	rs1190050	6	105849335	**0.0280***	NS
	rs1190053	6	105851881	**0.0361***	NS
FAM110A	rs552367	20	771504	**0.0102***	**0.013***
FAM110A_2	rs552367	20	771504	**0.0176***	NS

eSNPs: expression regulatory SNPs; *: $p < 0.05$; **NS:** Non-Significant;

Genevar: Gene Expression Variation, http://www.sanger.ac.uk/resources/software/genevar/.

Table S4. Results of the association tests between eSNPs and young-onset hypertension.

eSNPs	Allele	Chr.	Position	p-value
rs2916092	A/G	5	139394936	0.330
rs1051484	C/T	6	105832729	0.858
rs10871983	C/T	6	105942355	0.109
rs1149305	C/T	6	105852507	0.250
rs1149305	C/T	6	105852507	0.353
rs1149313	A/C	6	105860999	0.977
rs1190050	A/G	6	105849335	0.788
rs1190053	A/G	6	105851881	0.757
rs552367	G/T	20	771504	0.089

eSNPs: expression regulatory SNPs; Chr.: Chromosome.

Table S5. CNVs association test of the gene regions

Gene	Chr	Start	End	No. of Cases	No. of Controls	Empirical p-value
PREP	2	44399405	44440393	0	0	1
LAMB2	3	49133550	49145603	0	0	1
NRG2	5	139207443	139403063	0	0	1
ZRANB1	10	126620681	126665995	0	0	1
DCPS	11	125678856	125720854	0	0	1
ANKRD9	14	102042950	102045881	0	0	1
WFDC12	20	43185480	43186520	0	0	1
FAM110A	20	762355	774922	0	1	1
TPTE	21	9928613	10012791	0	0	1

Table S6. Association between nine genes and education, smoking and drinking.

Gene	Beta	SE	p-value	OR	95% CI	
**Education**						
ZRANB1	-0.04	0.20	0.859	0.97	0.65	1.43
DCPS	-0.18	0.18	0.293	0.83	0.59	1.17
NRG2	0.17	0.14	0.230	1.19	0.90	1.57
PREP	-0.01	0.18	0.967	0.99	0.70	1.40
WFDC12	0.23	0.18	0.187	1.26	0.89	1.79
FAM110A	0.22	0.14	0.132	1.24	0.94	1.65
LAMB2	0.24	0.24	0.306	1.28	0.80	2.04
TPTE	-0.11	0.21	0.611	0.90	0.60	1.36
ANKRD9	-0.25	0.12	0.044	0.78	0.61	0.99
**Smoking**						
ZRANB1	0.27	0.21	0.208	1.30	0.86	1.97
DCPS	-0.10	0.20	0.611	0.90	0.61	1.33
NRG2	0.13	0.15	0.397	1.14	0.85	1.53
PREP	0.17	0.20	0.377	1.19	0.81	1.75
WFDC12	0.34	0.20	0.089	1.41	0.95	2.08
FAM110A	0.01	0.16	0.962	1.01	0.73	1.39
LAMB2	0.41	0.28	0.139	1.50	0.88	2.59
TPTE	-0.02	0.21	0.933	0.98	0.66	1.47
ANKRD9	-0.29	0.14	0.040	0.75	0.57	0.99
**Drinking**						
ZRANB1	-0.23	0.18	0.199	0.79	0.55	1.13
DCPS	0.11	0.16	0.500	1.11	0.81	1.53
NRG2	0.26	0.13	0.043	1.30	1.01	1.68
PREP	0.02	0.16	0.902	1.02	0.74	1.40
WFDC12	0.05	0.16	0.768	1.05	0.76	1.44
FAM110A	0.36	0.14	0.013	1.43	1.08	1.89
LAMB2	0.14	0.22	0.523	1.15	0.75	1.78
TPTE	0.15	0.18	0.402	1.16	0.82	1.66
ANKRD9	0.13	0.11	0.269	1.14	0.91	1.42

Covariates, including age, sex, BMI, education, smoking, alcohol consumption, total cholesterol, HDL-C, s-GOT, s-GPT, eGFR, uric acid, and hypertension status were adjusted in the logistic regression model; SE: standard error; OR: odds ratio; CI: confidence interval.

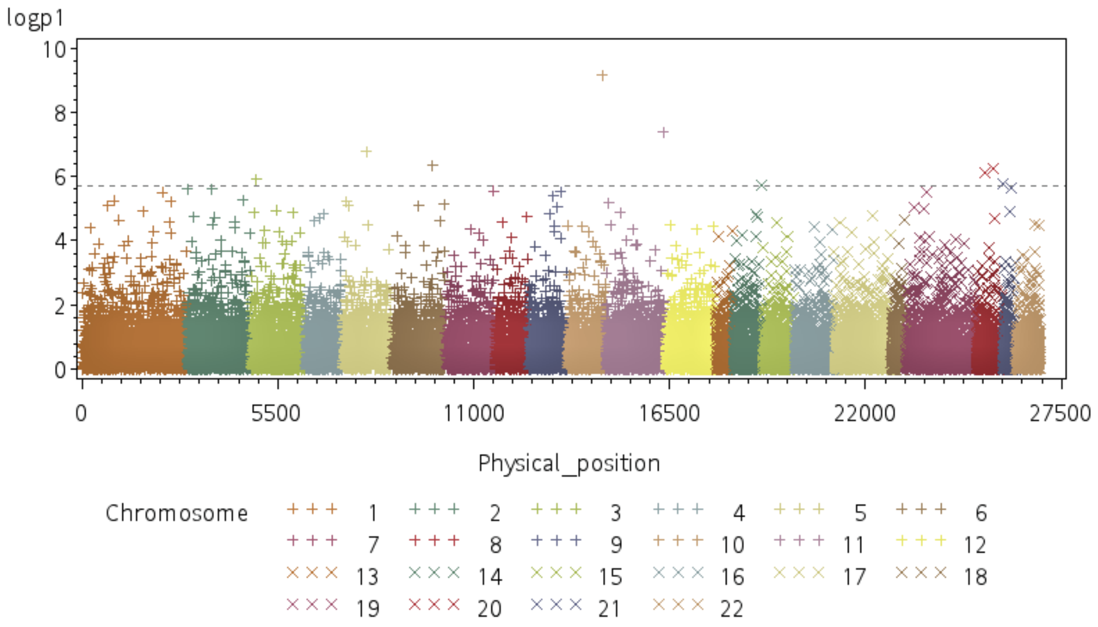

Figure S1. Manhattan plot of the first stage results. Dotted line indicates -log10 (p-value) = 5.7. [p-value = 1.98x10^{-6} (0.05/25215)].

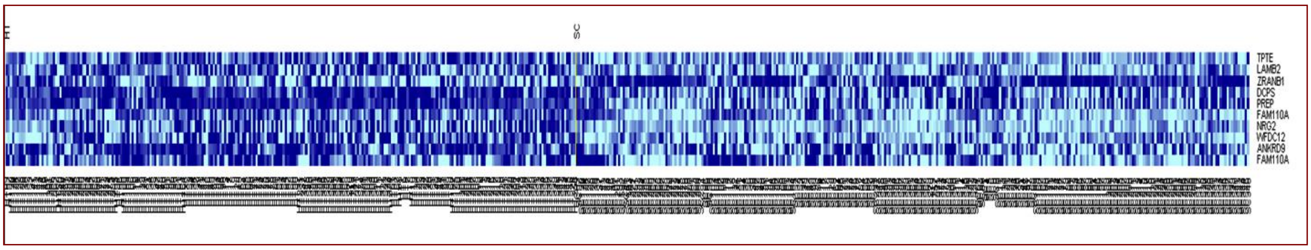

Figure S2. Heat plot for the nine genes identified by the 253 young-onset hypertension cases and 299 normotensive controls.
